# MSCs interaction with the host lung microenvironment: An overlooked aspect?

**DOI:** 10.3389/fimmu.2022.1072257

**Published:** 2022-11-15

**Authors:** Daniel J. Weiss, Sara Rolandsson Enes

**Affiliations:** ^1^ Department of Medicine, 226 Health Science Research Facility, Larner College of Medicine, University of Vermont, Burlington, VT, United States; ^2^ Department of Experimental Medical Science, Faculty of Medicine, Lund University, Lund, Sweden

**Keywords:** Mesenchymal stromal cells, MSCs, acute respiratory distress syndrome, cystic fibrosis, microenvironment, cell therapy, bronchoalveolar lavage fluid, lung

## Abstract

Mesenchymal stromal cells (MSCs) were identified more than 50 years ago, and research advances have promoted the translation of pre-clinical studies into clinical settings in several diseases. However, we are only starting to uncover the local factors that regulate cell phenotype, cell function, and cell viability across tissues following administration in different diseases. Advances in pre-clinical and translational studies suggest that the host environment, especially inflammatory active environments, plays a significant role in directing the infused MSCs towards different phenotypes with different functions. This can significantly effect their therapeutic efficacy. One way to study this interaction between the host environment and the infused cells is to expose MSCs *ex vivo* to patient samples such as serum or bronchoalveolar lavage fluid. Using this approach, it has been demonstrated that MSCs are very sensitive to different host factors such as pathogens, inflammatory cytokines, and extra cellular matrix properties. By understanding how different local host factors effect MSC function it will open possibilities to select specific patient sub-groups that are more likely to respond to this type of treatment and will also open possibilities to prime the local host environment to increase viability and to enrich for a specific MSC phenotype. Here, we aim to review the current understanding of the interaction of MSCs with the host microenvironment. To narrow the scope of this mini review, the focus will be on the pulmonary microenvironment, with a specific focus on the diseases acute respiratory distress syndrome (ARDS) and cystic fibrosis (CF).

## Introduction

Clinical studies investigating the effect of mesenchymal stromal cell (MSC)-based treatments for different lung diseases including acute respiratory distress syndrome (ARDS), chronic obstructive pulmonary disease (COPD), bronchopulmonary dysplasia (BPD), and cystic fibrosis (CF) have struggled with demonstrating significant improvement in outcomes (1–5). There are most likely multiple factors responsible for the lack of efficacy. Most focus so far has been on MSC characterization, cell origin, clinical outcomes, and the cell manufacturing process (6–8), there is less available information regarding the actual fate of the infused MSCs and what effect the diseased environment will have on the administered cells that end up in the target organ, in this case the lungs.

A better understanding on how the microenvironment, shaped by host factors and patient diversity, influence and direct MSC actions *in vivo* will help us to better understand the function of administered MSCs, design and execute better clinical trials, and increase the success rate of MSC-based therapies of severe lung diseases such as ARDS and CF. Here, we provide an overview of host microenvironmental factors in ARDS and CF patients that the administered MSCs encounter after infusion. These factors most likely influence the activity, viability, and function of the MSC-product and in combination with the intra- and interpatient microenvironmental variability it most likely contributes to the large differences in outcomes between pre-clinical and clinical studies.

In the first part of this review, we will discuss different host factors in ARDS, CF, and healthy lungs that influences MSC activity, and how clinical samples such as bronchoalveolar lavage fluid (BALF) and serum might help to over bridge at least parts of this knowledge gap. In the second part of this review, we will discuss different strategies to modulate the host environment.

## The diseased lung microenvironment and its effect on MSC function and viability

**Figure 1:**
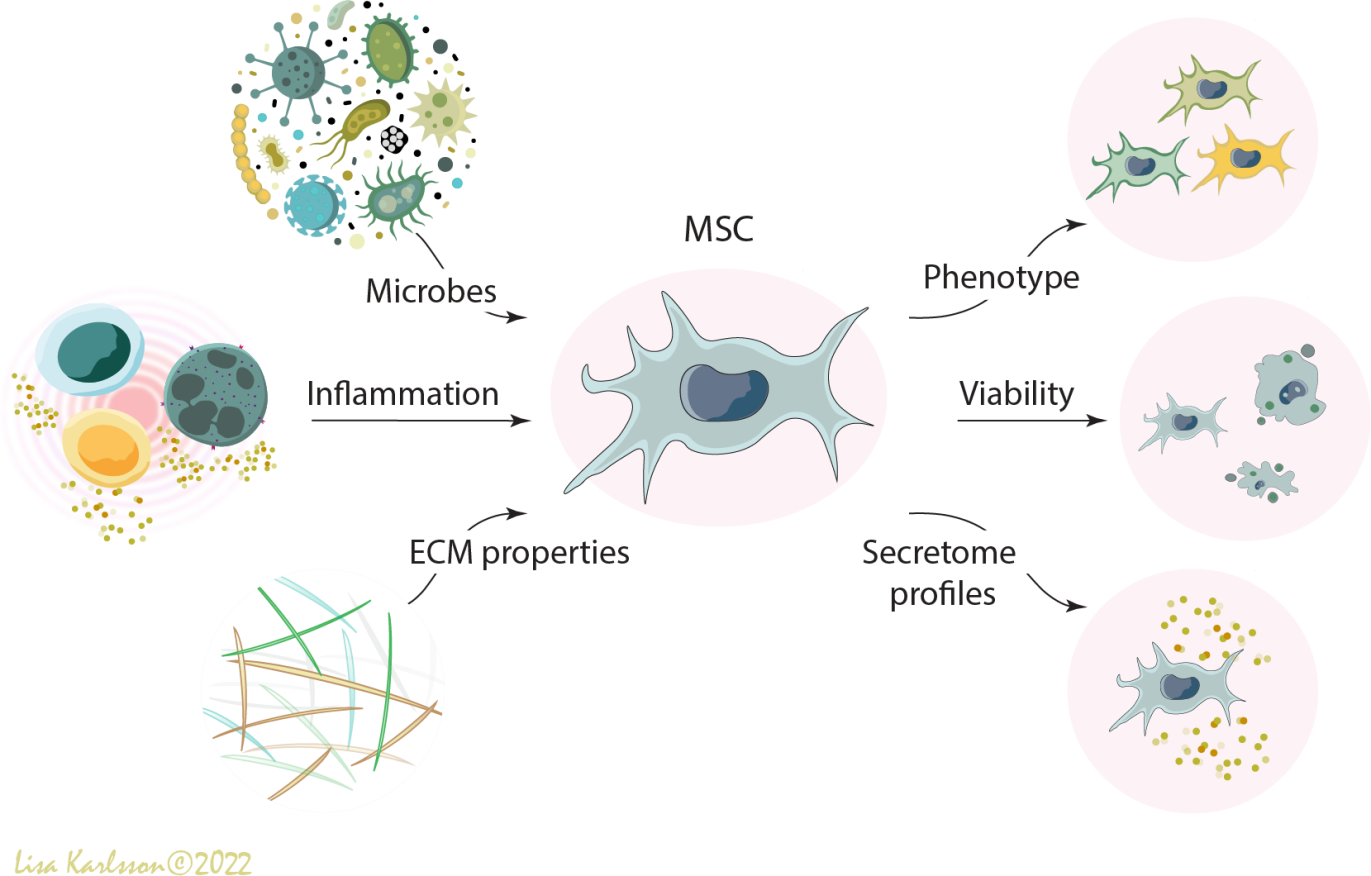
A schematic illustration describing how different environmental factors such as microbes (*e.g.* bacteria, virus, fungi, and PAMPs), inflammation (*e.g.* inflammatory cells and pro- and anti-inflammatory cytokines), and extracellular matrix properties (*e.g.* DAMPS, emphysema, fibrosis, elastic tendency of the lung, and oxygen tension) influence the viability, phenotype, and functions of administered human mesenchymal stromal cells (MSCs). MSC, mesenchymal stromal cells; PAMPS; pathogen-associated molecular patterns; DAMPS, danger-associated molecular patterns; ECM, extracellular matrix. Illustration created by Lisa Karlsson.

It is well known that cells interact with their local host environment and respond differently to different stress-signals including pathogen-associated molecular patterns (PAMPs) and danger-associated molecular patterns (DAMPs), released from adjacent cells, microbes, and/or the extracellular matrix [reviewed in (9–11)]. Severe pulmonary diseases such as ARDS and CF are inflammatory diseases where high levels of inflammatory cytokines, proteases, and other host compounds are present either locally in the lungs or systemically [reviewed in (12)]. In addition to the soluble factors that might affect MSC functions, the complexity of the lung structure such as the elastic tendency of the lung, and oxygen tension, are factors that most likely will further influence their behaviors (**Figure 1**). Other factors such as edema, fibrotic or emphysematous tissue, which are common complications in pulmonary diseases, might also alter MSC functions. For example, in *in vitro* settings it has been demonstrated that bone marrow derived MSCs differentiate towards different lineages depending on the stiffness and composition of the underlaying substrate (13, 14). All these different factors most likely contribute to the disappointing results in the completed clinical trials because they will all influence the function of the administered MSCs in different ways.

### The cystic fibrosis lung – a hypoxic host environment with viscous mucus and chronic inflammation

**Figure 2:**
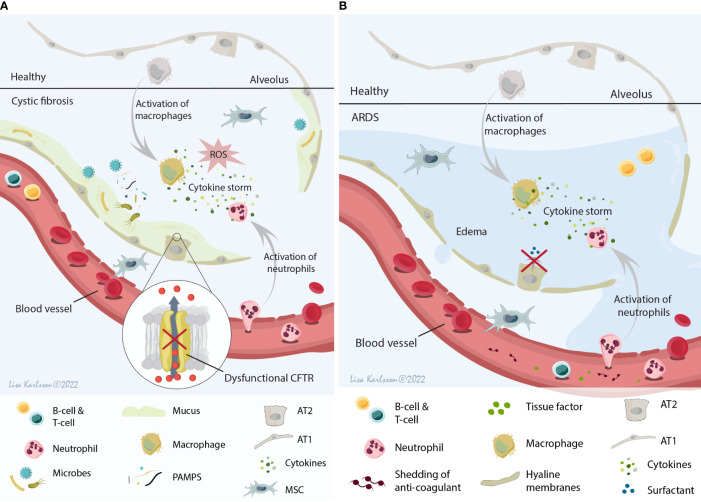
A schematic illustration summarizing the local host environment found in CF **(A)** and ARDS patients **(B)**. **(A)** The CF environment that MSCs encounter after infusion is a hypoxic and acidic environment with thick and sticky mucus that contribute to the increased risk of microbe colonization. This constant infection often results in a chronic inflammation with the release of several cytokines (IL-8 being one of the major cytokines), neutrophil influx, and ROS production leading to tissue damage and decreased lung function. **(B)** The ARDS environment is a protein rich and inflamed environment with activation of inflammatory cells and the release of pro-inflammatory cytokines (cytokine storm), often combined with injuries to both the alveolar and capillary barrier (which lead to edema). This result in paracellular permeability, impaired fluid clearance, impaired surfactant production, and shedding of anticoagulant molecules and tissue factors. MSCs, mesenchymal stromal cells; CF, cystic fibrosis; ARDS, acute respiratory distress syndrome; IL-8, interleukin 8; ROS, reactive oxygen species; CFTR, cystic fibrosis transmembrane conductance regulator; AT1, alveolar type 1 cells; AT2, alveolar type 2 cells. Illustration created by Lisa Karlsson.

The cystic fibrosis (CF) lung is a complex microenvironment that, due to the dysfunctional cystic fibrosis transmembrane conductance regulator protein (CFTR), is a hypoxic and acidic environment. Moreover, there is an imbalance in the water/chloride transportation resulting in an abnormal mucus consistency (reviewed in (15, 16)). This imbalance results in a thick and sticky mucus that contributes to an increased risk for infection and colonization of different microbes including *Staphylococcus aureus*, *Pseudomonas aeruginosa*, and *Aspergillus* (17, 18) for the majority of the CF patients. This continuing or repetitive microbe infections frequently lead to chronic inflammation, often an excessive neutrophilic inflammation with high levels of reactive oxygen species (ROS), high levels of circulating pro-inflammatory cytokines, and tissue damage (12, 19), creating a host environment that most likely will lead to a specific response and reaction if MSCs were to be infused into this milieu (**Figure 2A**). One of the major inflammatory players in CF pathophysiology is interleukin 8 (IL-8, also known as C-X-C motif ligand 8 (CXCL8)) which is a neutrophil chemotactic factor. Increased concentrations of IL-8 result in recruitment of additional neutrophils, but also stimulates phagocytosis which will lead to increased local levels of for example elastase and histamine (20–22).

The challenge around this issue lies, partly, in the difficulties to investigate the *in vivo* fate of administered MSCs. One possible way to increase the knowledge regarding the fate of infused MSCs into a CF host microenvironment is to expose MSCs *ex vivo* to patient samples including for example individual or pooled BALF and serum samples. Using this approach, we have previously demonstrated that human bone marrow derived MSCs exposed to individual BALF samples obtained from CF patients have an altered transcriptome and proteome profile compared to MSCs exposed to healthy control samples. A novel and important finding was that BALF samples from CF patients with an *Aspergillus* infection decreased MSC viability considerably, and the most likely responsible factor for the decreased viability was identified as the fungal mycotoxin Gliotoxin (23).

Taken together, these data suggest that also host pathogens might play an important role in the *in vivo* mode of action of MSCs. Suggesting that a better strategy to select patients for the MSC clinical trials, for example those without *Aspergillus* infection, in combination with a deeper understanding of how host microbes could alter the MSC therapeutic functions are needed.

### The acute respiratory distress syndrome lung – a leaky host environment with an aggressive immune response

Acute respiratory distress syndrome (ARDS) is a clinical syndrome with a complex pathophysiology that involves dysregulation and activation of multiple pathways of inflammation, coagulation, and injury in the lung. The multiple causes for the development of ARDS in combination with clinical and biological heterogeneity suggest that ARDS is an umbrella term that includes multiple phenotypes (24). These different phenotypes might be one of many factors responsible for the lack of improved outcome in clinical MSC trials of ARDS. Diffuse alveolar damage characterized by hyaline membrane deposition and neutrophilic alveolitis is one of the classical pathological findings in the ARDS lung identified post mortem in approximately 45% of the ARDS patients (25, 26). Moreover, injury to both the alveolar barrier and the capillary barrier is common in ARDS (25) and contributes to paracellular permeability, impaired fluid clearance [reviewed in (27)], impaired surfactant production (28), shedding of anticoagulant molecules and tissue factors (29, 30), and intensification of pro-inflammatory signaling [reviewed in (24)]. Taken together, these different pathophysiological findings in ARDS patients contribute to a complex inflammatory and severe edema host microenvironment (**Figure 2B**), that will most likely have a major effect on MSC biology and function. While pre-clinical models of acute lung injury may provide some information, they do not mimic the spectrum of clinical ARDS phenotypes and thus other approaches are needed.

One way to try to mimic this complex interaction of MSCs with the host environment is, as discussed above, to expose cells *ex vivo* to patient samples such as BALF or serum. Using this *ex vivo* model, we and others have demonstrated that MSCs respond or react differently to an ARDS environment compared to environments in other lung diseases, healthy lung, or control medium (23, 31–33). The exposure of pooled ARDS BALF samples to MSCs modulated macrophages by improving the phagocytic capacity of human monocytes, partly by transfer of mitochondria *via* CD44-mediated extracellular vesicles, compared to MSCs exposed to pooled healthy BALF samples (32). Moreover, MSCs exposed to serum from an experimental ARDS pig model demonstrated a changed secretome profile compared to MSCs exposed to serum obtained from untreated animals, with increased levels of IL-6, IL-1RA, IL-1β, IL-8, and IFN-γ (34). Similarly, an increased secretion of IL-6, IL-8, and IL-1β was observed by human MSCs exposed to human ARDS BALF samples compared to MSCs exposed to saline. No detection of IFN-γ was reported and IL-1RA was not measured in this study (33). The above-mentioned studies all focus on the inflammatory mediators and the effect on different immune cells. However, effects on MSCs of the other aspects of the ARDS pathophysiology needs to be thoroughly investigated in future studies.

### The healthy lung – the perfect host environment?

What about the healthy lung environment? Is the healthy lung a perfect environment that will allow MSCs to function ideally mirroring the results found in pre-clinical studies? Embedded in their native environment, MSCs are thought to be quiescent cells with low proliferation rate and high multi-potency (35). Moreover, in this inactive state MSCs have also been demonstrated to be mainly glycolytic cells with young mitochondria (35, 36).

Therefore, it is a bit surprising that MSCs exposed *ex vivo* to a healthy environment experienced modulation of their functional and phenotypical properties. For example, MSCs exposed to BALF samples obtained from healthy adult subjects demonstrated an altered secretome profile with increased secretion of pro-inflammatory mediator such as tumor necrosis factor (TNF), chemokine (C-X-C motif) ligand 10 (CXCL10), CCL2, CCL-8, interferon-beta1 (IFN-β1), and intercellular adhesion molecule 1 (ICAM1) compared to MSCs exposed to ARDS samples or phosphate-buffered saline control (33). Moreover, genes involved in self-recognition and coagulation including human leukocyte antigens (HLA)-DR and complements C3b, C4a, and C3A complement receptor (C3AR), was significantly upregulated in MSCs exposed to healthy BALF samples compared to ARDS samples or phosphate-buffered saline alone (33).

These data strongly suggest that in a healthy and stable lung an aggressive removal of external MSCs are induced by provoking an acute innate immune response, a mechanism similar to instant blood-mediated inflammatory reaction (37–39). As to why this aggressive removal occurs we can only speculate. One reason behind this could simply be that the cells are not needed in a healthy lung, and therefore are being recognized as foreign and effectively cleared from the lung. Interestingly, human MSCs that normally do not express HLA class II molecules (37, 40, 41), expressed increased levels of several HLA class II markers after exposure to BALF samples from healthy control subjects. Increased expression of HLA markers was not observed in MSCs exposed to ARDS samples, suggesting that MSCs may persist longer in an inflamed microenvironment compared to a non-inflamed milieu (33). These results parallel data from a recent pre-clinical study showing that MSCs infused into an *Escherichia coli*-infected mouse model were retained longer in the infected lung compared to the healthy non-infected lung (42). These observations further emphasize the great need for the basic understanding of the interaction between infused MSCs and the local host environment.

## Can we modulate the host environment to optimize the results of the MSC-based treatment?

An increased number of studies, including our own, demonstrate that MSCs react to and interact with local environmental factors such as microbes, PAMPS, inflammatory cytokines, and DAMPS (23, 31–33, 43). Parallel to the development of strategies for improving and standardizing the MSC cell-product we also need to start thinking of approaches on how, if possible, we can modulate or prime the host environment. Finding strategies to modulate the diseased lung (by *e.g.* adding or removing certain drugs) that will result in an environment that will be beneficial to MSCs and allow them to stay in the diseased lung for a longer period and at the same time keep their therapeutic phenotype would be ideal. This strategy has been tested in a totally different setting where it was demonstrated that priming a patient-derived xenograft mouse model of CD19+ B cell acute lymphoblastic leukemia with 5-azacytidine (AZA), a DNA methyltransferase inhibitor, resulted in delay of leukemia growth and promoted CAR T cell expansion (44). In addition, pre-treatment with low dose interferon-alpha was used to maintain the status of activated infused cells in an allogeneic stem cell transplantation (45). Moreover, pre-treatment with low dose interferon-alpha has been tested on patients with metastatic melanoma refractory prior adoptive cell therapy (ACT) treatment with promising results (46).

Interestingly, and in a totally different setting, a similar immune-priming strategy is being used by different pathogens to create harm and destabilize population dynamics resulting in pathogen persistence (47). One could think of using a similar strategy to create a favorable environment for the infused MSCs. One option that potentially can be beneficial is to skew the inflammatory response in the target lungs to favor activation of specific immune cells or to stimulate a local or systemic burst of specific cytokines prior MSC administration. This approach could potentially be applicable in patients that experiences more stable diseases, and not for example ARDS patients in the acute phase. Another approach, based on the knowledge discussed previously, could potentially be to give patients anti-fungal treatment to decrease the fungal load in the lungs before cell infusion, especially in CF patients which is a patient group that is more prone to fungal infections.

Another way would, instead of optimizing or modulating the host environment, be to select patients with a specific environmental profile for the MSC clinical trials. However, here we are dependent on the development of potency assays and/or discoveries of good biomarkers.

## Summary and final remarks

The fact that the host environment, in particular the inflammatory environment, affects cells and cell behavior is nothing new. However, this aspect has not been given enough attention and careful thought when optimizing and designing pre-clinical and clinical studies in the field of MSC-cell based therapies for severe diseases. This is in particularly important when considering MSC-based treatments for severe lung diseases since the lungs are constantly exposed to environmental factors and microbes. Moreover, patients with severe lung diseases often have active inflammation and persistent respiratory infections that will send out signals which interact with the infused cells. Today there is still very limited knowledge regarding the fate of infused MSCs, and how the cell viability and phenotypical and functional properties are altered after reaching, or even before reaching, the target organ. As discussed in this review, the main reason behind this lack of knowledge is the lack of good model system. However, lack of understanding and interest in the crosstalk between administered MSCs and the extra cellular matrix, factors and cells in the immune system, and/or pathogens, also contribute to this gap of knowledge. Using patient samples as a model to mimic the host environment *ex vivo* is a first step towards a better understanding of the *in vivo* fate of MSCs, but new model systems or animal models need to be developed. Equally important is that we start to assign more homogenous patient groups to the clinical trials, and perhaps also find ways to modulate or prime the patients before the treatment starts. Although this is no longer a young research field, rather a research field that has collected somewhat disappointing results from clinical trials for many years, we believe that an increased understanding of the interaction between the infused cells and the host microenvironment will lead to a significant progression in the field and improved clinical outcomes in future clinical trials.

## Author contributions

SRE and DW conceived the design, concept, and wrote the manuscript. All authors contributed to the article and approved the submitted version.

## Funding

This work is supported by Alfred Österlund Foundation, the Swedish Heart Lung Foundation, Per-Eric and Ulla Schyberg Foundation, the Crafoord Foundation, the Medical Faculty at Lund University, the Cystic Fibrosis Foundation, and the National Heart Lung Blood Institute of the US National Institutes of Health.

## Acknowledgments

The authors would like to acknowledge Lisa Karlsson at Lund University for drawing the illustrations included in this review.

## Conflict of interest

The authors declare that the research was conducted in the absence of any commercial or financial relationships that could be construed as a potential conflict of interest.

## Publisher’s note

All claims expressed in this article are solely those of the authors and do not necessarily represent those of their affiliated organizations, or those of the publisher, the editors and the reviewers. Any product that may be evaluated in this article, or claim that may be made by its manufacturer, is not guaranteed or endorsed by the publisher.
